# High Strength Concrete Columns under Axial Compression Load: Hybrid Confinement Efficiency of High Strength Transverse Reinforcement and Steel Fibers

**DOI:** 10.3390/ma9040264

**Published:** 2016-04-01

**Authors:** Wisena Perceka, Wen-Cheng Liao, Yo-de Wang

**Affiliations:** Department of Civil Engineering, National Taiwan University, No. 1, Sec. 4, Roosevelt Road, Taipei 10617, Taiwan; d02521014@ntu.edu.tw (W.P.); r02521242@ntu.edu.tw (Y.W.)

**Keywords:** column, confinement index steel fibers, confinement efficiency, effective confinement index, equivalent bond strength, high strength concrete, hybrid confinement, toughness ratio

## Abstract

Addition of steel fibers to high strength concrete (HSC) improves its post-peak behavior and energy absorbing capability, which can be described well in term of toughness. This paper attempts to obtain both analytically and experimentally the efficiency of steel fibers in HSC columns with hybrid confinement of transverse reinforcement and steel fibers. Toughness ratio (TR) to quantify the confinement efficiency of HSC columns with hybrid confinement is proposed through a regression analysis by involving sixty-nine TRs of HSC without steel fibers and twenty-seven TRs of HSC with hybrid of transverse reinforcement and steel fibers. The proposed TR equation was further verified by compression tests of seventeen HSC columns conducted in this study, where twelve specimens were reinforced by high strength rebars in longitudinal and transverse directions. The results show that the efficiency of steel fibers in concrete depends on transverse reinforcement spacing, where the steel fibers are more effective if the spacing transverse reinforcement becomes larger in the range of 0.25–1 effective depth of the section column. Furthermore, the axial load–strain curves were developed by employing finite element software (OpenSees) for simulating the response of the structural system. Comparisons between numerical and experimental axial load–strain curves were carried out.

## 1. Introduction

High strength concrete (HSC) has been increasingly used in reinforced concrete columns of high-rise buildings in recent years to further reduce member section size of the lower-story column and the concrete volume for the entire building structure [1,2]. Several researchers have reported, however, that the concrete turns more brittle if its compressive strength increases, and the confinement provided to HSC is less effective than in normal-strength concrete (NSC) [1,2,3]. Therefore, greater confinement in HSC columns is necessary to achieve similar strength and ductility enhancements [2,3]. In ACI 318-2014, additional transverse reinforcement is very much required for high strength concrete [4]. Adding short and discontinuous steel fibers can be an alternative to modify the brittle nature of HSC to ductile response in compression [1,2,5]. Several research studies showed that adding steel fibers to a NSC or HSC could improve its post-peak behavior and toughness [1,2,6,7,8,9,10,11,12,13]. This is because steel fibers provide bridging action across microcracks in the matrix and improve resistance to crack opening due to the existence of the bond strength between the steel fibers and the matrix [1]. Several experimental studies have been undertaken to more fully understand the compressive behavior of HSC columns with hybrid confinement of hooked-end steel fibers and transverse reinforcement [2,7,14,15,16]. However, not enough works are available in the literature, apart from a research study by Paultre *et al.* [2] who proposed an effective confinement index from the hybrid confinement of transverse reinforcement and steel fibers. Thus limited information was available on the hybrid confinement efficiency of HSC columns in compression with transverse reinforcement and steel fibers. In this study, an equation for quantifying hybrid confinement efficiency of transverse reinforcement and steel fibers in HSC columns is proposed by regressing test results obtained by previous researchers, expressed in term of transverse reinforcement and steel fiber parameters, and verified by using test results obtained from compression tests carried out in this study.

## 2. Research Significance

This study attempts to obtain both analytically and experimentally the efficiency of steel fibers as confinement in HSC columns with hybrid confinement of transverse reinforcement and steel fibers under axial compression load, since less information on the comprehensive study of HSC columns with hybrid confinement was available. In an analytical study, the toughness ratio (TR) equation was proposed, expressed in terms of transverse reinforcement and steel fiber parameters, and was derived through a regression analysis by involving 69 TRs of HSC columns without steel fibers with concrete compressive strength ranging from 53 MPa to 124 MPa, and 27 TRs of HSC column hybrids of transverse reinforcement and steel fibers with concrete compressive strength and fiber volume fractions ranging from 68 MPa to 200 MPa and 0.25% to 1.5%, respectively. Using TR to quantify confinement efficiency is more feasible than using nominal axial strength column (P_o_) since TR expresses the material energy absorbing capability which is associated with post-peak behavior, while P_o_ generally expresses the maximum strength of the column and has no relationship with its ductility. Compression tests on 17 HSC columns with different fiber volume fractions and transverse reinforcement ratios were conducted as well, where high strength longitudinal and transverse reinforcement were provided for 12 specimens. The results from the compression tests were used to verify the proposed regression analysis. In addition, the presence of the bond strength of steel fibers and matrix (equivalent bond strength) is an important steel fiber parameter besides fiber length, fiber diameter, and fiber volume fraction [1]. Steel fiber pullout tests were also conducted to compute the equivalent bond strengths, and then verified with a micromechanical model proposed by Xu *et al.* [17].

The influence of fiber volume fractions, transverse reinforcement ratios, and high strength steel on the maximum axial load, strain corresponding to peak load, the shape of load-displacement curve, and the TR are discussed. The proposed TR is compared with TRs obtained from experimental results, and an example calculation to compute the equivalent confinement from a hybrid of transverse reinforcement and steel fibers is provided in Appendix A. In order to carry out a cross comparison study of axial load-strain curves of HSC columns, an experimental and numerical, finite element application software (OpenSees) [18] for simulating the response of the structural system was employed.

## 3. Proposed Analytical Model for Toughness Ratio of HSC Columns

**Figure 1:**
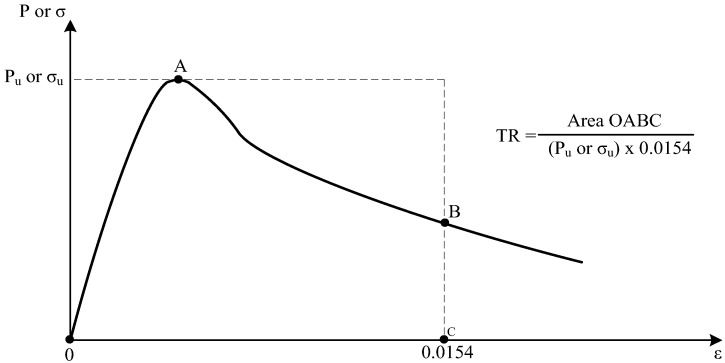
Definition of toughness ratio (TR).

Toughness can be defined by the area under the stress-strain curve or load-strain curve [6], and the toughness ratio is defined as the ratio of the toughness of a concrete specimen to the toughness of the rigid material [6], as given in Figure 1. Fanella and Naaman [19] reported that the compression tests on fiber mortar cylinders were not continued beyond a strain of about 0.0154, in which the specimens still had residual strengths; the toughness of the specimen was calculated up to a strain of 0.0154 and thus led to lower bound estimate of the toughness. In this study, the ultimate strain used in calculating toughness is 0.0154, and Equation (1) is given to compute toughness ratio *TR*.

(1) $$TR=\frac{\mathrm{area}\mathrm{under}\mathrm{the}\mathrm{force}-\mathrm{strain}\mathrm{curve}\mathrm{up}\mathrm{to}\varepsilon \mathrm{of}0.0154}{P_{\text{max}}\times 0.0154}$$

As previously mentioned, the TR expressed in term of transverse reinforcement and steel fiber parameters is proposed to account confinement efficiency, because *TR* expresses an energy absorbing capability which is associated with post-peak behavior.

### 3.1. TR Prediction Model for HSC Confined by High Strength Transverse Reinforcement

**Figure 2:**
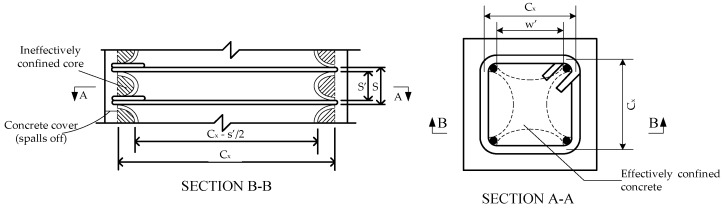
Arching action in confined concrete and effective confined core.

According to Cusson and Paultre [20], for HSC confined by high strength transverse reinforcement, the confinement index is generally expressed in terms of the volumetric ratio of transverse reinforcement in concrete core, $\rho_{h}$, the yield stress of transverse bar, $f_{yh}$, and the concrete compressive strength, ${f\prime }_{c}$. The confinement effectiveness coefficient, $K_{e}$, used by Mander *et al.* [21] was also adopted by Cusson and Paultre [20], because the maximum lateral pressure from the confining steel could only be exerted effectively on the part of the concrete core where the confining stress had fully developed due to arching action (Figure 2 [20,21]).

**Figure 3:**
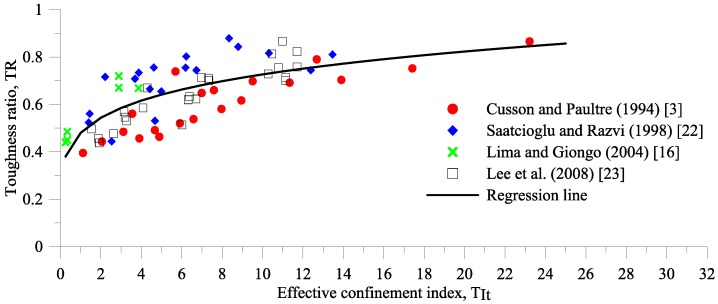
The relationship of TR and effective confinement index $k_{e}\rho_{s}f_{\text{yt}}/{f\prime }_{c}$.

The TR prediction equation [(Equation (2)] for HSC confined by transverse reinforcement is expressed in term of effective confinement index, *T*_It_, and was derived by regressing 69 experimental results obtained from compression tests on HSC columns with high strength transverse reinforcements [3,16,22,23], as shown in Figure 3. The concrete compressive strengths and the transverse reinforcement yield strengths range from 53 to 124 MPa and 392 to 1000 MPa, respectively.

(2) $${\text{TR}}_{\text{HSC},\text{prediction}}=0.48{\left(T_{It}\right)}^{0.18}$$

The following expression is given to calculate the effective confinement index, *T*_It_, and the confinement effectiveness factor, k_n_:

(3) $$T_{\text{It}}=\frac{k_{e}\cdot \rho_{h}\cdot f_{\text{yh}}}{{f\prime }_{c}k_{n}}$$

and

(4) $$k_{n}=\frac{n_{l}}{n_{l}-2}$$

in which *k*_n_ is the confinement effectiveness factor and *n_l_* is the number of longitudinal bars around the perimeter of the column core which are laterally supported by the corner of hoops or by conventional ties with 135° hooks anchored in the core, and $\rho_{h}$ is expressed in percent. The confinement effectiveness *k_n_* is provided and required in ACI Code 318-2014 chapter 18 [4] for computing the required confinement.

### 3.2. HSC Columns with Hybrid Confinement of Transverse Reinforcement and Steel Fibers

#### 3.2.1. Confinement Index of Steel Fibers in HSC

The previous researches showed that the TR of HSC with steel fibers significantly depended on the fiber volume fraction, *V_f_*, and the aspect ratio of fibers, *l_f_*/*d_f_*, [6,7,12,13]. However, the presence of bond strength between steel fiber and matrix (equivalent bond strength) τ_eq_ was neglected in most studies. Liao *et al.* [1] reported that by considering the bond strength between steel fibers and matrix and treating steel fibers as confinement, the post-peak behavior of concrete material could be well-described. The confinement index steel fibers, *T*_If_, in concrete, is given in the following equation:

(5) $$T_{If}=V_{f}\frac{l_{f}}{d_{f}}\times \frac{\tau_{\text{eq}}}{{f\prime }_{\text{cf}}}$$

where $V_{f}$ is expressed in percent, the unit system of *τ*_eq_ and *f*’_cf_ are MPa, and *f*’_cf_ is the cylinder strength of concrete with steel fibers. The *τ*_eq_ is calculated by using a micromechanical model for the pullout energy of hooked-end steel fiber in cement-based composites proposed by Xu *et al.* [17].

#### 3.2.2. Effective Confinement Index from Hybrid of Transverse Reinforcement and Steel Fibers

This paper is the first study to express TR in terms of transverse reinforcement and steel fiber parameters; the confinement index equation for HSC column hybrids of stirrups and steel fibers has been proposed by Paultre *et al.* [2]. Paultre *et al.* [2] proposed the hybrid confinement index ${I\prime }_{e}$ for a square section as simply superposition from transverse confinement and steel fibers, as given in Equation (6).

(6) I′e=ρse·f′hf′c+ηθτufvf(lfdf)f′c

where $\rho_{se}=$ effective sectional ratio of the confining reinforcement perpendicular to either the x- or y-axes, which is equal to half of the effective volumetric lateral reinforcement ratio 12keρh; $\eta_{\theta }=$ fiber orientation efficiency factor of 3/8; $\tau_{uf}=$ assumed as constant frictional bond strength. For the rectangular section, parameter $\rho_{\text{se}}$ in the first part of Equation (6) can be calculated by using the equation proposed by Cusson and Paultre in 1995 [20]. Paultre *et al.* [2] proposed the transverse steel reinforcement stress at concrete peak stress, ${f\prime }_{h}$, for fiber reinforced concrete confined by transverse reinforcement as follows:

(7) f′h={fyh0.25f′c+10ηθτufvf(lfdf)ρse(κ−10) ≥0.43 ε′cEs≯fyh

Parameter ${\varepsilon \prime }_{c}$ and ${\text{E}}_{s}$ are strains corresponding to peak stress of plain concrete and modulus elasticity of steel, respectively. Parameter κ is to determine whether the lateral reinforcement yields at the peak strength of confined concrete, as given in Equation (8).

(8) $$\kappa =\frac{{f\prime }_{c}}{\rho_{se}E_{s}{\varepsilon \prime }_{c}}$$

**Figure 4:**
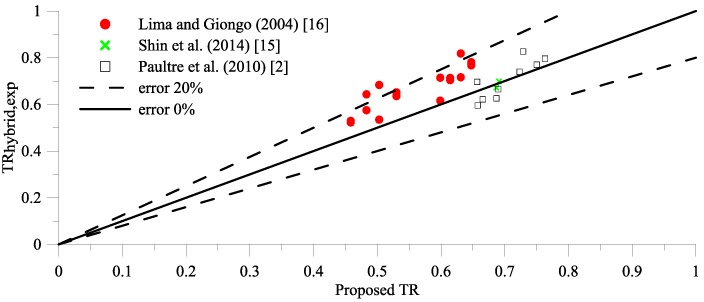
The error between TR_hybrid,exp_ and proposed TR with hybrid confinement index ${I\prime }_{e}$.

Substituting Equation (6) into proposed TR_HSC,prediction_ [Equation (2)] to calculate TR_hybrid,prediction_, however, tends to produce errors between proposed TR_hybrid,prediction_ and TR_hybrid,exp_ larger than 20%, as illustrated in Figure 4. In order to minimize errors (Figure 4), TR_hybrid,prediction_ will be expressed in terms of effective confinement index, *T_It_*, and fiber factor, $x_{f}$, as given in Equation (9).

(9) $${\text{TR}}_{\text{hybrid},\text{prediction}}=0.48{\left(x_{f}\cdot T_{\text{It}}\right)}^{0.18}$$

**Figure 5:**
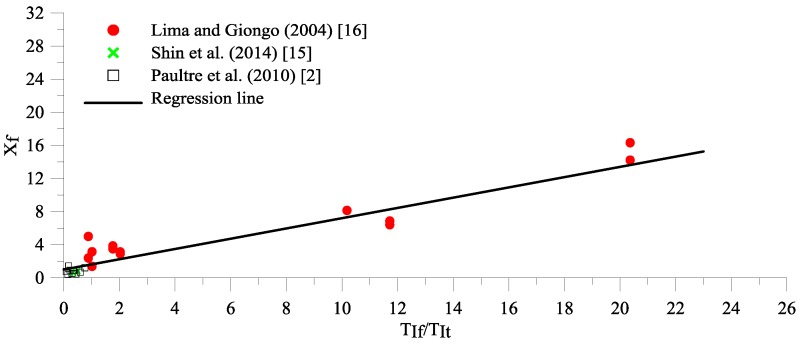
The relationship of fiber factor $x_{f}$ and TIfTIt.

The fiber factor, $x_{f},$ must be influenced by the ratio of the confinement index steel fibers to the effective confinement index (TIfTIt) in HSC. By substituting every value of TR HSC columns of hybrid transverse reinforcements and steel fibers from many test results into Equation (2), the effective confinement index equivalent, $T_{\text{It},\text{equivalent}}$, can be obtained, while the fiber factor, $x_{f}$, is the ratio $T_{It,equivalent}$ to $T_{\text{It}}$, (TIt,equivalentTIt). The relationship between fiber factor, $x_{f}$, and (TIfTIt) is plotted in Figure 5. Regression analysis was conducted on 27 experimental data of HSC column hybrids of transverse reinforcements and steel fibers [2,15,16] with concrete compression strengths from 68 to 200 MPa, yield strengths and transverse reinforcement volumetric ratios from 410 MPa to 856 MPa and 0.61% to 6.09%, respectively, and fiber volume fractions from 0.25% to 1.5%. The fiber factor, $x_{f}$, is given in Equation (10).

(10) $$x_{f}=1+0.62\frac{T_{If}}{T_{It}}$$

#### 3.2.3. TR Prediction Model for HSC Column Hybrids of Transverse Reinforcement and Steel Fibers 

It can be observed from Figure 5 that the fiber factor $x_{f}$ increases if the confinement index of the transverse reinforcement decreases and the confinement index of the steel fibers increases. Therefore, the steel fibers are more effective when the spacing transverse reinforcement becomes larger. The proposed TR given in Equation (11) is for HSC confined by transverse bars and steel fibers, and obtained by substituting Equation (10) into Equation (9).

(11) $$TR_{\text{hybrid},\text{prediction}}=0.48{\left(T_{It}+0.62T_{If}\right)}^{0.18}$$

**Figure 6:**
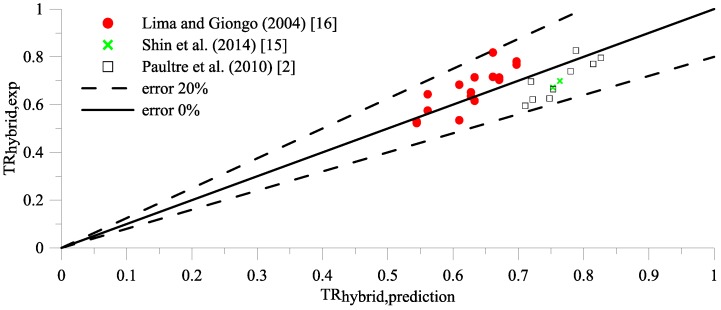
The error between TR_hybrid,exp_ and TR_hybrid,prediction_.

By using Equation (11), the greatest error is less than 20%, as shown in Figure 6.

## 4. Experimental Program

In order to verify the proposed TR_hybrid,prediction_ relationship, an experimental program was carried out, which is discussed in the following sections.

### 4.1. Material and Mixing Procedure

**Figure 7:**
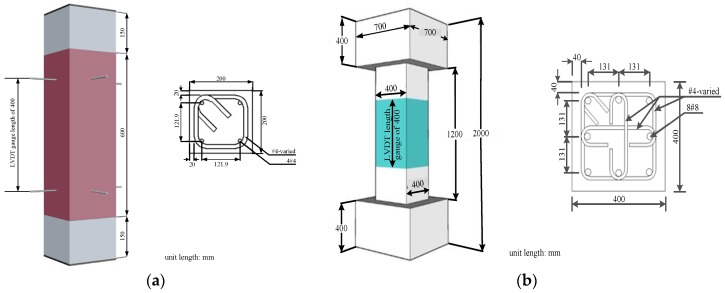
Detail of the tested specimens: (**a**) Small-scale column; (**b**) Large-scale column.

The test program involved 9 square small-scale and 8 square large-scale steel fiber HSC columns, which were subjected to axial compression load. The small-scale and large-scale column sizes were 200 mm × 200 mm × 900 mm and 400 mm × 400 mm × 1200 mm, respectively. Three different fiber volume fractions (0.75%, 1%, and 1.5%) were used, and the volumetric ratios of transverse reinforcement in the concrete core varied between 0% and 7.92%. The details of the test specimens and bar configurations are shown in Figure 7.

**Figure 8:**
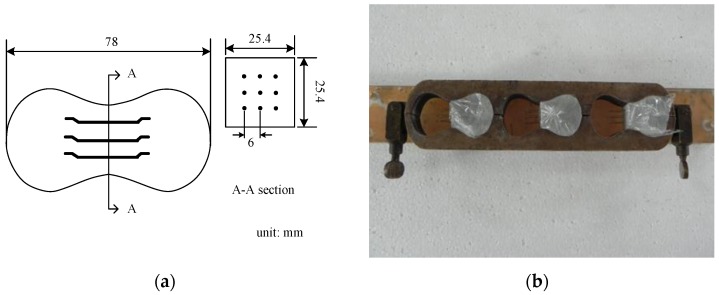
(**a**) The section of and shape of mortar specimen for fiber pullout tests; (**b**) special shape mold.

The design concrete strength, f’_c_, was 100 MPa for small scale columns and 70 MPa for large-scale columns and the concrete mix proportions are summarized in Table 1. The mix proportions for small-scale columns were very similar to that used by Liao *et al*. [1]. Portland cement type 1 with specific gravity of 3.15, granulated blast furnace (GGBS) slag with specific gravity of 2.82, silica fume with specific gravity of 2.21, coarse aggregates of maximum size of 9.5 mm, natural river sand, and two superplasticizers with specific gravity of 1.08 ± 0.01 and 1.035–1.075 were used in this study. The steel fibers used were Dramix-RC-80/30-BP with tensile strength of 2300 MPa, diameter of 0.38 mm, and a length of 30 mm. The mixing procedure was in accordance with the mixing procedure for self-consolidating high performance fiber reinforced concrete proposed by Liao *et al.* [24]. In Table 1, the codes used for the specimens are as follows: for small scale columns, the uppercase N or H denotes either normal or high strength rebars with the first number after either capital N or H and the last number denoting transverse reinforcement spacing in mm and fiber volume fraction in percent, respectively; for large scale columns, S is defined as word spacing, and is followed by the transverse reinforcement spacing in mm and the fiber volume fraction in percent. The nine specimens to conduct fiber pullout tests were produced using the mix proportion which was exactly the same as the mix proportions used for casting small-scale columns with a fiber volume fraction of 1.5%. The specimens with a rectangular cross section and nine hooked steel fibers embedded in the middle section of each specimen were cast in special shape molds, as shown in Figure 8.

For N-series small scale columns, the bars with yield strengths of 280 MPa were selected for longitudinal reinforcement, and the bars with yield strengths of 420 MPa were selected for transverse reinforcement. The bars with yield strength of 785 MPa were used as longitudinal and transverse reinforcement in H-series small-scale columns. The bar size #4 with diameter of 12.7 mm was used in all series of small-scale columns. For large-scale columns, the bar size #8 with diameter of 25.4 mm and yield strength of 685 MPa was selected for longitudinal reinforcement, and the bar size #4 (diameter of 12.7 mm) with yield strength of 785 MPa was selected for transverse reinforcement. There was a concrete block at both the top end and the bottom end of each large-scale column; therefore, the total height of each large-scale column was 2000 mm. For each small-scale column, a steel plate with thickness of 6 mm was provided at the top end and bottom end of the column to obtain the uniform stress on the column section. At least three reinforcing bars from each specified yield strength were taken to undergo the tensile test so that the average actual yield strength could be obtained. Table 2 presents both the specified yield strength and the average actual yield strength of bars, longitudinal and volumetric transverse reinforcement ratios, and fiber volume fractions for each specimen.

### 4.2. Nominal Axial Strength Design of the Column Specimen

The nominal axial strength of the column at zero eccentricity (P_o_) of all column specimens was in accordance with the ACI ITG-4.3 R-07 [25] procedure, where the design expression is shown in Equation (12).

(12) $$P_{o}=x_{1}f_{c}^{\prime }\left(A_{g}-A_{st}\right)+f_{y}A_{st}\;$$

where *A_g_* = total cross-sectional area of column; *A_st_* = total cross-sectional area of longitudinal reinforcement; *f_y_* = yield strength of longitudinal bar; $x_{1}$ is a factor which can be taken as 0.85 for normal strength concrete, which is reduced continuously at a rate of 0.0022 for each MPa of strength in excess of 55 MPa, but it cannot be less than 0.7.

### 4.3. Testing Procedure

**Figure 9:**
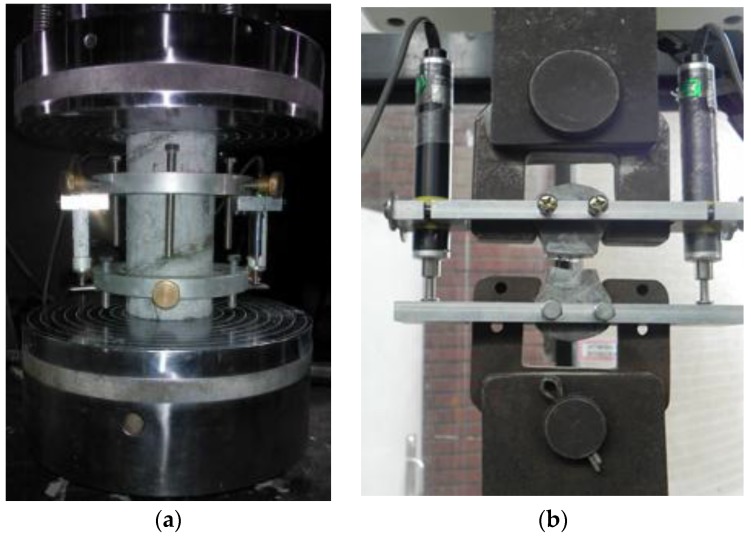
Test setup: (**a**) compression tests on concrete; (**b**) fiber pullout tests.

The concrete cylinders were tested on a servo-hydraulic closed-hoop testing machine with a capacity of 1000 kN which applied a monotonically increasing displacement loading at a constant rate of 0.01 mm/s. Two LVDTs (Linear Variable Displacement Transducers) provided on both sides of each concrete recorded the concrete axial strains, as shown in Figure 9a. The compression tests on concrete cylinders were conducted when testing the corresponding column specimens. A servo-hydraulic closed-loop testing machine with capacity of 30 kN and displacement rate of 0.01 mm/s was employed to conduct the fiber pullout tests. In order to measure the fiber pullout slip, two LVDTs were arranged to obtain the complete fiber pullout load and slip curves as shown in Figure 9b.

**Figure 10:**
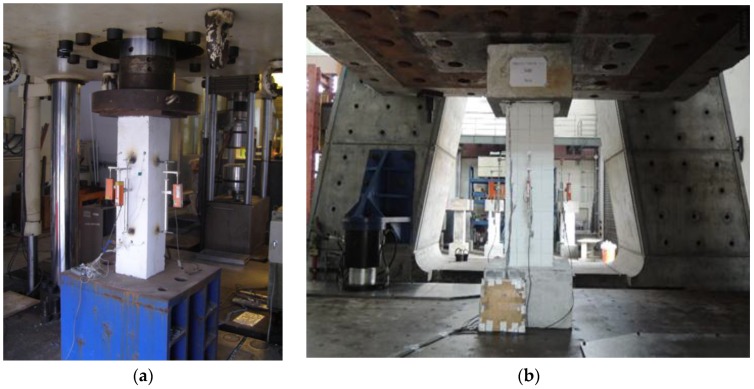
Test setup of column specimen subjected to axial compression load: (**a**) Small-scale column; (**b**) Large-scale column.

All columns were subjected to concentric axial compression load. The compression tests on all series of small scale columns were conducted under a servo-hydraulic closed-hoop testing machine with a capacity of 5000 kN, where the displacement rate of the machine was 0.0375 mm/s. For the measurement of the steel strain, two electrical strain gauges were laid on one longitudinal steel bar, and there were three strain gauges on one transverse bar (close hoop). These strain gauges were located around the mid-height of column. Four LVDTs with a gauge length of 400 mm recorded the axial strains, and were located at side of and at the mid-height of each column specimen. The large-scale columns were tested in the National Center Research for Earthquake Engineering (NCREE) Taiwan, by using the Multi-axial Resting System (MATS). At least, three electrical strain gauges on one longitudinal steel bar and transverse bars (one on the close hoop bar and two on ties) were available to measure the steel strains in the concrete, and were located around the mid-height of the column. MATS can apply a maximum axial load of 60 MN, and the loading rate employed was 0.05 mm/s. Figure 10 shows the test setup for a small-scale and large scale column.

### 4.4. Experimental Results and Discussion

Table 3 presents the test results, including concrete cylinders compressive strength, f’_co_, nominal axial strength at zero eccentricity, P_o_, maximum axial load carried by the concrete column, P_c,max,exp_, strain corresponding to the peak load, ε_c,max,exp_, ratio of P_c,max,exp_ to P_o_, and toughness ratio, TR_exp_, obtained from the column specimens.

#### 4.4.1. Compression Strength Test and Fiber Pullout Test

**Figure 11:**
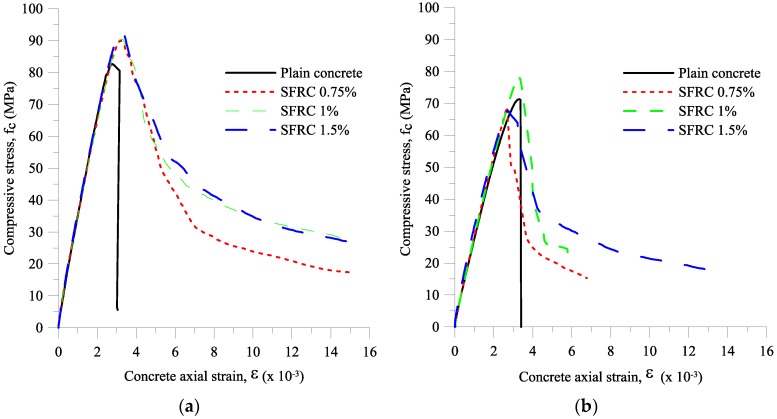
Stress-strain curve of high strength concrete: (**a**) from batch for small-scale columns; (**b**) from batch for large-scale columns.

**Figure 12:**
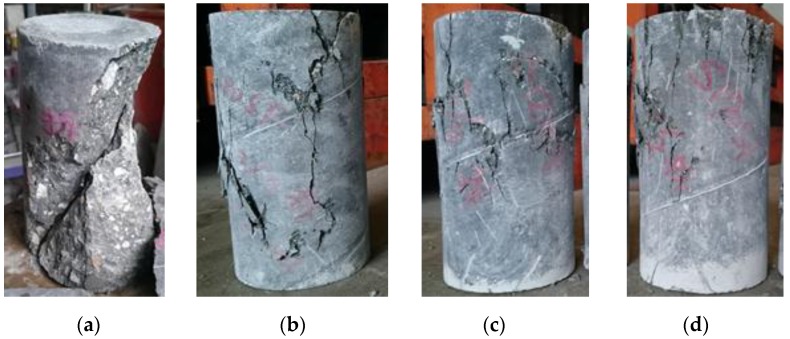
The failure modes of concrete specimens with: (**a**) *V_f_* of 0%; (**b**) *V_f_* of 0.75%; (**c**) *V_f_* of 1%; (**d**) *V_f_* of 1.5%

The concrete axial strain was the average strain obtained from the two LVDTs provided at each of the column specimens. Figure 11 shows the stress-strain curve of the concrete cylinders and Figure 12 shows the failure modes of the cylinders with different fiber volume fractions. It can be seen in Figure 11 that adding steel fibers to plain concrete improved its post-peak response. The damages on plain concrete specimens were splitting and shear cone, and the specimens with fibers showed cracks around the edge and middle of the specimens, as shown in Figure 12.

**Figure 13:**
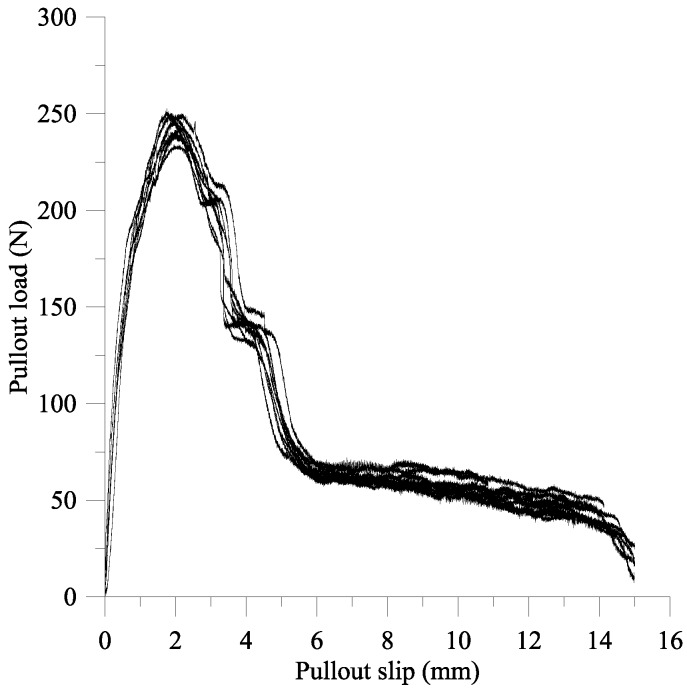
Nine pullout load-slip curves.

Figure 13 shows the pullout load-slip curves obtained from fiber pullout tests, and the area under the pullout load-slip curve shows the pullout energy E_pullout_. The equivalent bond strength, τ_eq_, can be defined from Equation (13) [26].

(13) $$\tau_{eq}=\frac{2\cdot E_{pullout}}{\pi \cdot d_{f}\cdot L_{embedded}^{2}}$$

where *d_f_* = diameter of the fibers; *L_embedded_* = length of the fiber embedded into the matrix. The analytical model for computing the pullout energy proposed by Xu *et al.* [17] is employed to verify the results obtained from fiber pullout tests. The average pullout energy in this experimental study was 1408.05 N mm, which was only 9.8% difference from that calculated based on the progressive micromechanical model for the pullout energy of hooked-end steel fiber in cement-based composites proposed by Xu *el al.* [17]. By using Equation (13), the equivalent bond strength τ_eq_ produced from fiber pullout tests and from the analytical model were 10.49 MPa and 11.52 MPa, respectively. The comparison between Xu’s model [17] and the fiber pullout tests was also conducted by Liao *et al.* [1] for matrix strength ranging from 30 MPa to 60 MPa, where the greatest error between results from Xu’s model and test results was 13%. Accordingly, Xu’s model [17] is valid for further estimation of the equivalent bond strength *τ*_eq_ of steel fiber in concrete without providing a fiber pullout test.

#### 4.4.2. Compression Tests of Small Scale Columns

**Figure 14:**
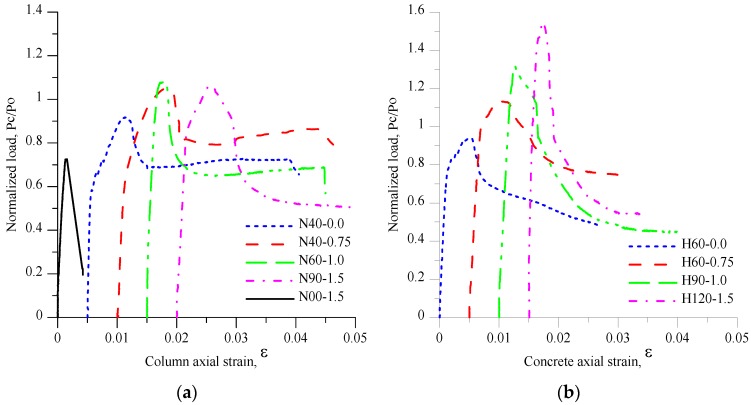
The normalized load *P_c_ /P_o_ versus* concrete axial strain curve *ε* for small-scale column specimens: (**a**) N-series; (**b**) H-series.

Figure 14 presents the normalized axial compression load–axial strain curves for small-scale columns, where the normalized axial load is defined as the ratio of the axial load carried by the concrete to the axial strength design of unconfined concrete (PcPo). As summarized in Table 3, the maximum axial load carried by small-scale concrete columns ranged from 2334 kN to 3569 kN, and the normalized axial compression load PcPo varied between 0.73 and 1.13. Either the highest PcPo or the highest *P_c_*_,max,exp_ was obtained from specimen H60-0.75. Table 3 also presents the strain corresponding to the peak load ε_c,max,exp_ which varied between 0.0015 and 0.008. The peak strain *ε_c,_*_max,exp_ obtained from specimen N40-0.75 was the highest peak strain if compared with peak strains obtained from other small-scale columns specimens, while the *ε_c_*_,max,exp_ of specimen H60-0.75 was the second highest. As seen in Figure 14a,b, the slope of experimental curves (PcPo-axial strain curves) changed before the peak point was reached due to cracking of the concrete cover and yielding of longitudinal reinforcement. At this stage, it could be estimated that the crack opening was getting larger and leading to spalling or crushing of the concrete cover while the axial strain increased. For column specimens without steel fibers, the concrete core strength improved due to the passive confinement pressure corresponding to the lateral expansion of the concrete. For column specimens with steel fibers, the concrete core improved due to both the passive confinement pressure and the steel fibers bridging action. However, for specimen N00-1.5, the axial capacity of the column significantly dropped once the peak point was reached.

**Figure 15:**
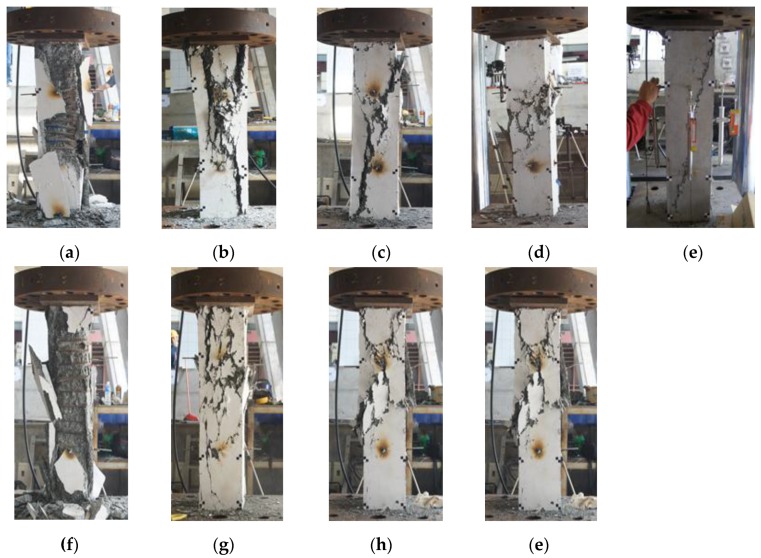
The final appearance of small-scale columns after testing: (**a**) N40-0.0; (**b**) N40-0.75; (**c**) N60-1.0; (**d**) N90-1.5; (**e**) N00-1.5; (**f**) H60-0.0; (**g**) H60-0.75; (**h**) H90-1.0; (**i**) H120-1.5.

For specimens N40-0.0, N40-0.75, N60-1.0, the axial load dropped to around 77%, 78%, and 63% of the peak load, respectively, due to the buckling of longitudinal reinforcement, and the post peak loads of these three specimens approaching constant value. The pre-peak behavior (ascending branch) of specimen N90-1.5, H60-0.0, H60-0.75, H90-1.0, and H120-1.5 was similar to that of specimen N40-0.0, N40-0.75, and N60-1.0; however, the post peak load decreased slowly, and did not approach constant value, as shown in specimens N40-0.0, N40-0.75, and N60-1.0. In general, the descending branch existed due to the ductile behavior of the concrete core. Figure 15 presents the types of damages of small-scale columns. For specimens without steel fibers (N40-0.0 and H60-0.0), the concrete cover almost totally spalled off (Figure 15a,f). It can be concluded that specimens N40-0.0 and H60-0.0 were well-confined specimens. The other well-confined specimens were N40-0.75 and H60-0.75, in which many cracks appeared on the concrete cover of the two specimens at the failure stage. This was because adding fibers to concrete improved the concrete material by avoiding strain localization due to the bridge effect from the steel fibers. Moreover, the post peak loads on the descending branch of specimens N40-0.75 and H60-0.75 were not only greater than those of N40-0.0 and H60-0.0 but also greatest among the post peak loads obtained from other small-scale columns tests.

The specimens H90-1.0 and H120-1.5 were low-confined specimens, because the concrete cover of these two specimens did not show more cracks at the failure stage. Furthermore, the post-peak loads on the descending branch up to a strain of 0.03 of specimens H90-1.0 and H120-1.5 were much lower than those of specimens N40-0.0, N40-0.75, H60-0.0, and H60-0.75. The specimens N60-1.0 and N90-1.5 can be categorized as moderate-confined specimens. As seen in Figure 14, the post-peak loads on the descending branch of specimen N60-1.0 tended to approach constant value up to a strain 0.03 and were greater than those of specimen N90-1.5, H90-1.0, H120-1.5. For specimen N90-1.5, the post-peak behavior was more ductile than the post-peak behavior of specimen H90-1.0 and H120-1.5.

**Figure 16:**
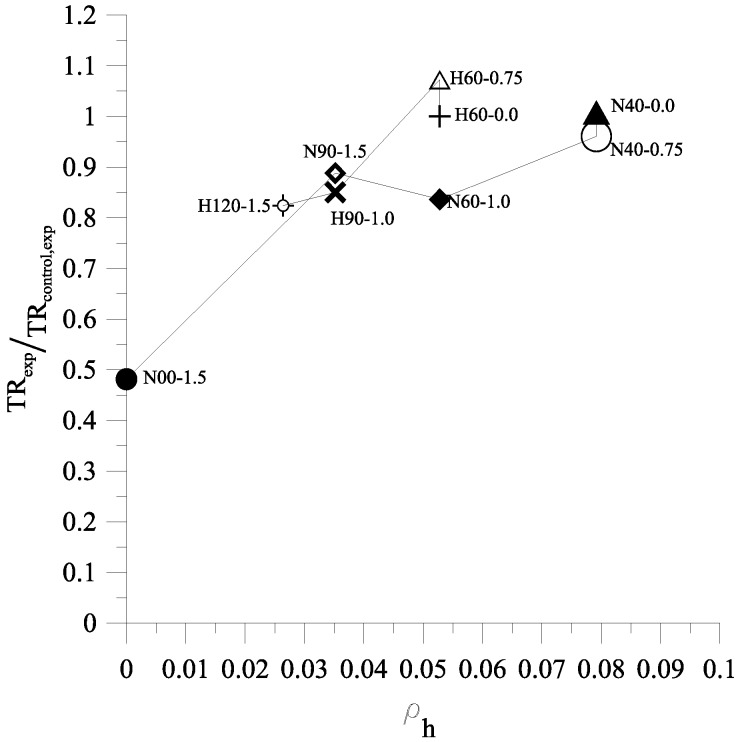
The relationship of TR_exp_/TR_control,exp_ and ρ_h_ for small-scale columns.

The experimental toughness ratios, *TR*_exp_, are summarized in Table 3. Figure 16 shows the relationship of TRexpTRcontrol,exp and volumetric ratio of transverse reinforcement in the concrete core $\rho_{h}$, in which $TR_{control,exp}$ is the TR of control specimens (N40-0.0 and H60-0.0). As shown in Figure 16, for N-series, the $\rho_{h}$ varied between 0 and 0.079 while TRexpTRcontrol,exp ranged from 0.48 to 1. For H-series of small-scale columns, when $\rho_{h}$ ranged from 0.026 to 0.053, TRexpTRcontrol,exp varied between 0.82 and 1.

Furthermore, it could be observed that the use of high strength steel in the HSC column under a concentric axial compression load improved the maximum axial load carried by the column. As shown in Figure 14, the maximum axial loads of the H-series specimens were larger than those of the N-series specimens. However, in the TR case, TR was not only influenced by the high strength steel but also the presence of fiber and the volumetric ratio of the transverse reinforcement in the concrete core, $\rho_{h}$. It can be seen in Table 3, for N-series columns, that the TR ranged from 0.67 to 0.8 (excluding specimens N00-1.5), while the TRs of the H-series specimens ranged from 0.63 to 0.82.

#### 4.4.3. Compression Tests of Large Scale Columns

**Figure 17:**
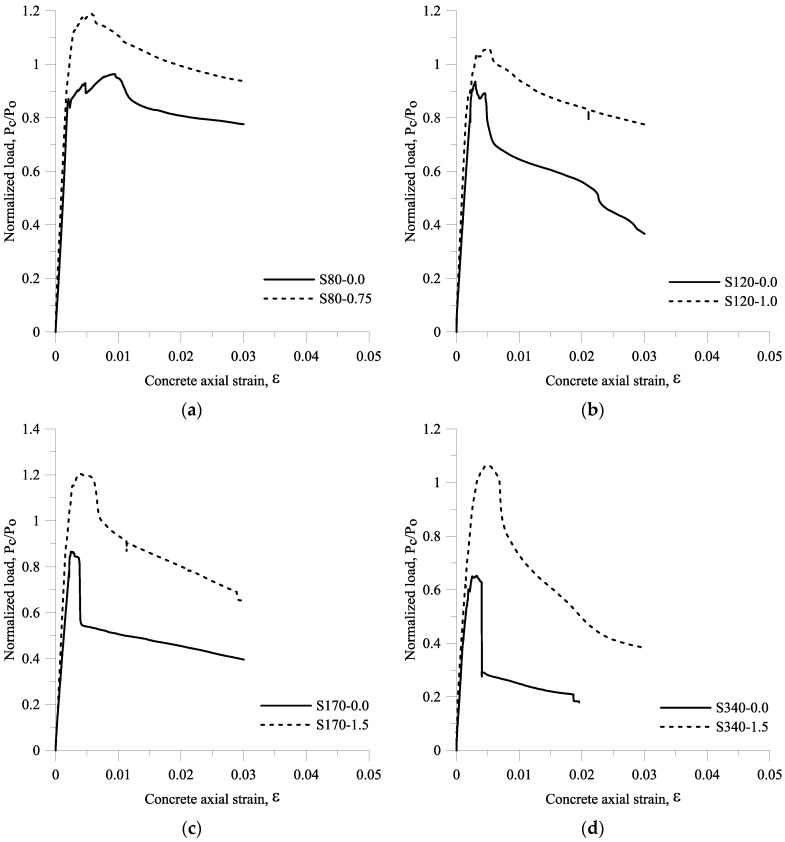
The normalized load *P_c_/P_o_ vs.* concrete axial strain curve ε for large-scale column specimens: (**a**) S80-0.0 and S80-0.75; (**b**) S120-0.0 and S120-1.0; (**c**) S170-0.0 and S170-1.5; (**d**) S340-0.0 and S340-1.5.

**Figure 18:**
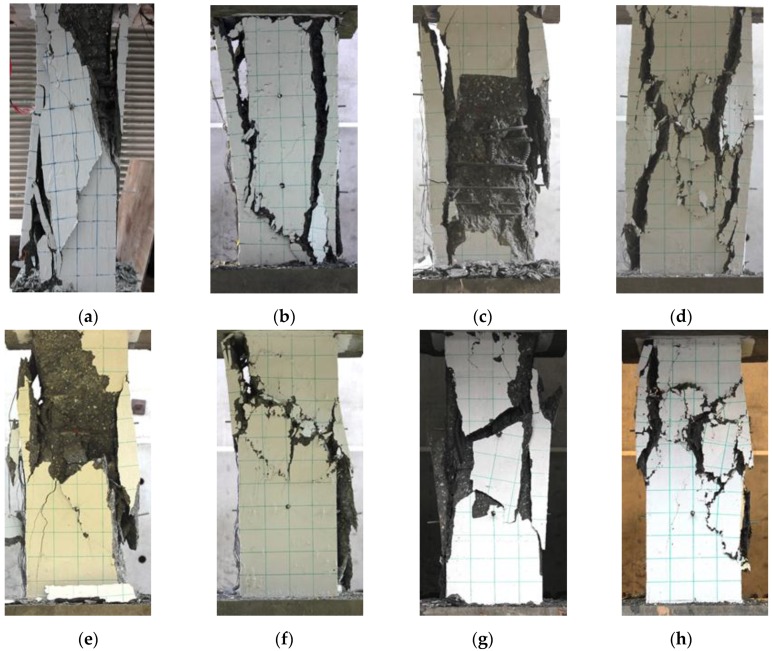
The final appearance of large-scale columns after testing: (**a**) S80-0.0; (**b**) S80-0.75; (**c**) S120-0.0; (**d**) S120-1.0; (**e**) S170-0.0; (**f**) S170-1.5; (**g**) S340-0.0; (**h**) S340-1.5.

Figure 17 shows the PcPo–axial strain curves and Figure 18 shows the failure modes of the specimens for large-scale columns. The maximum axial load carried by the concrete column ranged from 7703.8 kN to 13,634.9 kN, and the normalized axial compression load PcPo varied between 0.65 and 1.2. As summarized in Table 3, the strain corresponding to the peak load ε_c,max,exp_ varied between 0.0025 and 0.0061. The peak strain *ε_c,max,exp_* obtained from specimen S80-0.0 was the highest peak strain if compared with other peak strains obtained from other large-scale columns tests, while the *ε_c,max,exp_* of specimen S80-075 was the second highest. As presented in Figure 17a through Figure 17d, the slope of the ascending branch on PcPo–axial strain curves obtained from specimen S80-0.0, S80-075, S120-1.0, S170-1.5, and S340-1.5 also changed before the peak load was reached due to cracking of the concrete cover.

For specimens S80-0.0, S80-0.75 and S120-1.0, the post peak loads decreased slowly. For specimens S120-0.0, S170-0.0, and S340-0.0, the axial load tended to be straight from a load of 0 up to the first peak load, dropped to around 90% of the maximum axial load and then reached the second peak load directly. As presented in Figure 17b through Figure 17d, the axial load dropped to around 78%, 65%, and 43% of the second peak load for specimen S120-0.0, S170-0.0, and S340-0.0, respectively, due to buckling of the longitudinal reinforcement, and was then followed by the descending branch. The ascending branch of specimens S170-1.5 and S340-1.5 was similar to that of specimen S80-0.0, S80-0.75, and S120-1.0; however, the axial load dropped to around 79% of the peak load due to the buckling of the longitudinal reinforcement, and was then followed by the descending branch.

The specimen S80-0.0 was a well-confined specimen, because the concrete core failed after most of the concrete cover spalled off, as shown in Figure 18a. The specimen S80-0.75 was also a well-confined specimen. This is because the post peak loads of S80-0.75 were greater than those of S80-0.0, and although the concrete cover disintegrated from the concrete cover (at failure point), some of the concrete covers still remained standing, as shown in Figure 18b. The specimen S120-0.0 can be categorized as a moderate-confined specimen. It can be seen from Figure 18c that the failure stage corresponded with the spalling of the concrete cover at the middle and bottom of the column. The presence of fibers with a volume fraction of 1% in S120-1.0 resulted in the concrete showing more cracks on its cover at the failure stage. In addition, the post peak loads of specimen S120-1.0 were greater than those of specimen S120-0.0. The specimen S170-0.0 and S340-0.0 were poorly confined specimens. As seen in Figure 18e,g, S170-0.0 and S340-0.0 failed before all the concrete covers spalled off, and the longitudinal bar of these two specimens buckled. The presence of steel fibers in S170-1.5 and S340-1.5 improved its post-peak behavior, as seen in Figure 17c,d. Figure 18f,h shows that the concrete covers of specimens S170-1.5 and S340-1.5 did not totally disintegrate from their core at the failure stage.

**Figure 19:**
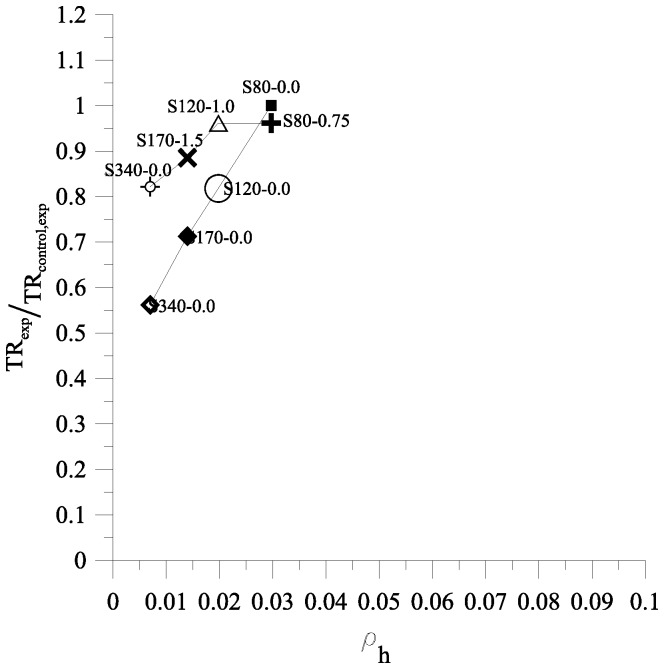
The relationship of TR_exp_/TR_control,exp_ and ρ_h_ for large-scale colums

The relationship of TRexpTRcontrol,exp and $\rho_{h}$ for large-scale columns is presented in Figure 19. For specimens without fibers, $\rho_{h}$ ranging from 0.007 to 0.03 corresponded with TRexpTRcontrol,exp ranging from 0.562 to 1. By contrast, for specimens with fibers, $\rho_{h}$ ranging from 0.007 to 0.03 corresponded with TRexpTRcontrol,exp ranging from 0.82 to 0.96. As seen in Figure 19, the steel fibers had significant influence on TR when spacing transverse reinforcement increased. First, it can be observed from TRexpTRcontrol,exp of specimen S80-0.75 which is very close to 1, where this result showed the presence of steel fibers has little effect. This result is also observed on specimens N40-0.0 and H60-0.0 from small column tests. Secondly, it can be observed in Figure 19 that TRexpTRcontrol,exp of specimens S120-0.0, S170-0.0, S340-0.0, S120-1.0, S170-1.5, and S340-1.5 are equal to 0.82, 0.71, 0.56, 0.96, 0.88, and 0.82, respectively. Therefore, the presence of steel fibers increases the TR by 20%, 26%, and 49% for columns with transverse reinforcement spacing of 120 mm, 170 mm, and 340 mm, respectively.

### 4.5. The Transverse Reinforcement Spacing in Column Hybrids of Transverse Reinforcement and Steel Fibers

According to the test results, the presence of steel fibers is more effective when the transverse reinforcement spacing becomes larger. However, as shown by Figure 14a, the specimen N00-1.5 (no transverse reinforcement) was brittle even though the fiber volume fraction was 1.5%. In addition, the compression behavior of specimen S340-1.5 with transverse reinforcement spacing of d was better than that of S340-0.0. Therefore, the transverse reinforcement spacing in a column hybrid of transverse reinforcement and steel fibers should be in the 0.25 to 1 effective depth d of the member section (0.25d to d).

### 4.6. Verification of TR Prediction Model for HSC Column Hybrid of Transverse Reinforcement and Steel Fibers

The compression test results of HSC column hybrids of transverse reinforcement and steel fibers from Lima and Giongo [16], Paultre *et al.* [2], and the authors (only test results from HSC columns with hybrid confinement) are used to verify the validation of Equation (11). Seventeen specimens with concrete compressive strength ranging from 65.1 MPa to 101.40 MPa, transverse reinforcement ratio varying between 0.61% and 7.29%, and fiber volume fractions ranging from 0.25% to 1.5% have been verified. All the design details and parameters needed for Equation (11) are summarized in Table 4. The TRs obtained from experimental results and those calculated using Equation (11) are summarized in Table 4 as well. The errors for 17 specimens are less than 20%. Therefore, Equation (11) is valid for further *TR* estimation for HSC column hybrids of transverse reinforcement and steel fibers, and can also be applied for HSC without steel fibers if *T_If_* is equal to zero for HSC without steel fibers (Equation (11) will be exactly the same as Equation (2)).

## 5. Cross Comparison Study of the Numerical Model and Experimental Results

**Figure 20:**
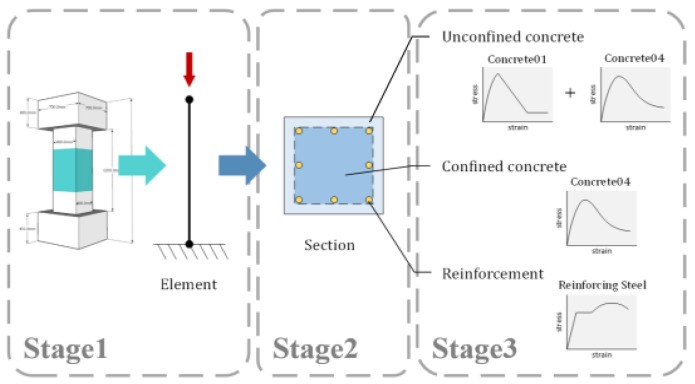
Stress-strain relationship of concrete column and longitudinal reinforcement in the modeling process.

Developing axial load–axial strain curves from the numerical model was conducted in order to verify whether the use of TR in term of transverse reinforcement and steel fiber parameters is feasible to obtain confinement efficiency. OpenSees (Open System for Earthquake Engineering Simulation) [18], a finite element application software for simulating the response of structural and geotechnical systems subjected to earthquake was employed to develop axial load-axial strain curves of column hybrids of stirrups and steel fibers. The steel fibers need to be converted into transverse reinforcements to obtain the equivalent effective confinement index T_It_,_equivalent_ since no software provides a stress-strain model for concrete confined by transverse reinforcement and steel fibers. The stress-strain model for confined concrete was based on the model proposed by Paultre *et al*. [2]. Figure 20 shows the procedure conducted in the modeling column specimen, and the example calculation provided in Appendix A is to present the use of the proposed TR model in obtaining the equivalent effective confinement index for HSC column hybrids of transverse reinforcement and steel fibers.

**Figure 21:**
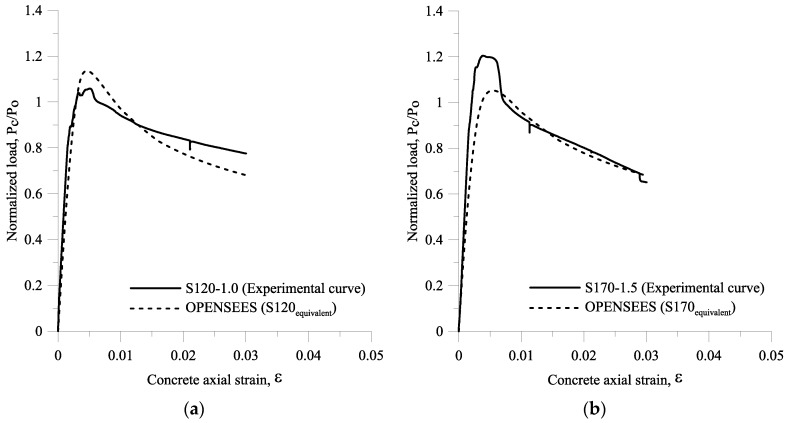
Comparison between the normalized load *P_c_/P_o_*–axial strain from experimental curves and the normalized load *P_c_/P_o_*–axial strain from the numerical model for large scale columns for: (**a**) S120-1.0; (**b**) S170-1.5.

The cross comparison study of experimental load-strain curves and load-strain curves developed by using the numerical model is illustrated in Figure 21. The specimens S120-1.0 and S170-1.5 were selected as case studies. The results obtained from the load-strain curves are summarized in Table 5. From Figure 21, it can be observed that the curves developed by using the analytical model have good agreement with those obtained from the experimental program. In addition, as presented in Table 5, two of the TRs from the numerical models are exactly the same as those from the experimental results. The average error between the experimental results and the numerical results is 6.1%. Therefore, the TR model expressed in term of transverse reinforcement and steel fiber parameters is generally valid to determine the confinement efficiency of HSC column hybrids of transverse reinforcement and steel fibers. Since the steel fibers are treated as confinement, the equivalent bond strength $\tau_{\text{eq}}$ is an important parameter, and should be considered in the analysis or design of an HSC column with steel fibers. Furthermore, using another analytical software and following the procedure shown in Figure 20 produces similar results.

## 6. Conclusions

This paper presents an analytical study of and a series of compressive tests on HSC column hybrid confinement of transverse reinforcement and steel fibers under axial compression load. Based on the results of this study, the following conclusions can be drawn:

1. Adding steel fibers to HSC columns can improve their post peak behavior. This is because steel fibers provide a bridge effect due to the presence of the bond strength between the steel fibers and the matrix (equivalent bond strength).
2. In this study, not only the fiber volume fraction and aspect ratios, but also the equivalent bond strength *τ_eq_* should be considered in accounting for fiber characteristics. The test results obtained from fiber pullout tests were verified using a progressive micromechanical model proposed by Xu *et al.* The average pullout energy in this experimental study was only 9.8% difference from that calculated by using the micromechanical model proposed by Xu *et al*. in 2011, where the equivalent bond strength *τ_eq_* can be computed by using the equation proposed by Kim *et al.* in 2007. Accordingly, *τ_eq_* can be calculated based on the progressive micromechanical model if no fiber pullout test results are provided.
3. From the test results of small-scale columns, it can be observed that a TR of a specimen with high strength steel rebars may be similar to that of a specimen with normal strength rebars. This is because *TR* is not only influenced by the grade of rebars but also by the transverse reinforcement spacing and the fiber volume fractions.
4. A *TR* equation for HSC confined by transverse reinforcement is proposed based on the regression analysis on 69 TRs for HSC columns confined by transverse reinforcement.
5. The *TR* for HSC confined by transverse reinforcement (*TR*_HSC,prediction_) was modified by involving fiber factor $x_{f}$. TRs of the columns hybrid confinement of transverse reinforcement and steel fibers from the test results are substituted into *TR*_HSC,prediction_ equation to obtain the effective confinement index equivalent $T_{It,equivalent}$, and the fiber factor $x_{f}$ is the ratio $T_{It,equivalent}$ to $T_{\text{It}}$, xf=(TIt,equivalentTIt). By employing regression analysis on 27 TRs of column hybrid of transverse reinforcement and steel fibers, the fiber factor $x_{f}$ can be expressed in term of the ratio of the effective confinement index of the transverse reinforcement $T_{It}$ to the confinement index of steel fibers, $T_{If}$. The proposed TR can be applied for HSC columns with a concrete compressive strength and fiber volume fraction ranging from 65 MPa to 200 MPa and 0% to 1.5%, respectively.
6. The efficiency of steel fibers in concrete depends on the spacing of the transverse reinforcement, in which the steel fibers are more effective if the spacing transverse reinforcement becomes larger. The relationship of fiber factor $x_{f}$ and TIfTIt shows that the fiber factor $x_{f}$ becomes larger when TIfTIt increases. It should be noted that this is only for the transverse reinforcement spacing ranging from d/4 to d.
7. The column specimen with no transverse reinforcement is still brittle even though the fiber volume fraction is 1.5%, as observed on specimen N00-1.5.
8. The errors between $TR_{hybrid}$ from experimental tests and $TR_{hybrid}$ prediction are less than or equal to 20%.
9. Employing toughness ratio TR in term of transverse reinforcement and steel fiber parameters is feasible to compute the confinement efficiency of HSC column hybrids of transverse reinforcement and steel fibers. This is because TR expresses the energy absorbing capability, in which the energy absorbing capability is associated with post-peak behavior.
10. Based on the transverse reinforcement equivalent, the confinement model proposed by Paultre *et al.* in 2010 was adopted to develop the stress-strain model for concrete confined in OpenSees. The results obtained from OpenSees well-match the results obtained from compression tests.
11. The proposed *TR* in this study should be further verified through HSC column hybrids of transverse reinforcement and steel fibers subjected to high axial compression load and reversed cyclic loading.

## Figures and Tables

**Table 1:** Mix proportions of concrete matrices (kg/m^3^).

Specimens		*Vf* (%)	Cement	GGBS Slag	Silica fume	Water	Course aggregate	Sand	Steel fiber	SP
Type	ID									
Small-scale column (200 mm × 200 mm × 900 mm)	N40-0.0	0.00	365	285	50	164	866	647	0	4.5
	H60-0.0									
	N40-0.75	0.75	362	283	50	163	860	642	59	4.5
	H60-0.75									
	N60-1.0	1.00	361	282	50	162	857	640	79	4.5
	H90-1.0									
	N00-1.5 N90-1.5	1.50	360	281	49	161	853	637	118	4.5
	H120-1.5									
Large-scale column (400 mm × 400 mm × 1200 mm[clear height])	S80-0.0	0.00	400	170	50	165	844	719	0	14.9
	S120-0.0									
	S170-0.0									
	S340-0.0									
	S80-0.75	0.75	382	262	51	220	410	1026	59	8.8
	S120-1.0	1.00	378	260	51	218	407	1017	79	8.9
	S170-1.5	1.50	372	255	50	215	400	1000	118	9.3
	S340-1.5									

**Table 2:** Details of column specimens.

ID	*bc* = *hc* (mm)	Longitudinal Reinforcement						Transverse Bars								Steel Fiber
		Bar Size (No.)	*f_y_* (MPa)	*f_y_*_,test_ (MPa)	*n*	*ρ_cc_* (%)	*w*′ (mm)	Bar Size (No.)	*f_yt_* (MPa)	*f_yt_*_,test_ (MPa)	*s* (mm)	*ρ_h_ (%)*	*C_x_* = *C_y_* (mm)	*k_e_*	*V_f_*(%)	*L_f_*/*D_f_*
N40-0.0	200	4	280	335	4	2.34	109.20	4	420	478	40	7.92	147.30	0.53	0.00	−
N40-0.75	200	4	280	335	4	2.34	109.20	4	420	478	40	7.92	147.30	0.53	0.75	79
N60-1.0	200	4	280	335	4	2.34	109.20	4	420	478	60	5.28	147.30	0.46	1.00	79
N90-1.5	200	4	280	335	4	2.34	109.20	4	420	478	90	3.52	147.30	0.35	1.50	79
H60-0.0	200	4	785	774	4	2.34	109.20	4	785	774	60	5.28	147.30	0.46	0.00	−
H60-0.75	200	4	785	774	4	2.34	109.20	4	785	774	60	5.28	147.30	0.46	0.75	79
H90-1.0	200	4	785	774	4	2.34	109.20	4	785	774	90	3.52	147.30	0.35	1.00	79
H120-1.5	200	4	785	774	4	2.34	109.20	4	785	774	120	2.64	147.30	0.26	1.50	79
N00-1.5	200	4	280	335	4	−	134.60	−	−	−	−	−	−	−	1.50	79
S80-0.0	400	8	685	703	8	4.29	109.20	4	785	886	80	2.97	307.30	0.76	0.00	−
S80-0.75	400	8	685	703	8	4.29	109.20	4	785	886	80	2.97	307.30	0.76	0.75	79
S120-0.0	400	8	685	703	8	4.29	109.20	4	785	886	120	1.98	307.30	0.65	0.00	−
S120-1.0	400	8	685	703	8	4.29	109.20	4	785	886	120	1.98	307.30	0.65	1.00	79
S170-0.0	400	8	685	703	8	4.29	109.20	4	785	886	170	1.40	307.30	0.53	0.00	−
S170-1.5	400	8	685	703	8	4.29	109.20	4	785	886	170	1.40	307.30	0.53	1.50	79
S340-0.0	400	8	685	703	8	4.29	109.20	4	785	886	340	0.70	307.30	0.21	0.00	−
S340-1.5	400	8	685	703	8	4.29	109.20	4	785	886	340	0.70	307.30	0.21	1.50	79

**Table 3:** Test Results.

ID	*Vf* (%)	*f*′_co_ (MPa)	*x_1_*	*P_o_* (kN)	*P_c_*_,max,exp_ (kN)	*ε_c_*_,max,exp_ (mm/mm)	*P_c_*_,max,exp_/*P_o_*	*TR*_exp_
N40-0.0	0.00	86.80	0.79	2837.67	2602.50	6.25E-03	0.92	0.80
H60-0.0		88.20	0.78	2871.40	2701.00	5.36E-03	0.94	0.76
S80-0.0		72.50	0.82	11991.24	11551.58	9.00E-03	0.96	0.87
S120-0.0		70.50	0.82	11781.02	11018.70	3.02E-03	0.94	0.71
S170-0.0		70.50	0.82	11781.02	10201.90	2.50E-03	0.87	0.62
S340-0.0		70.80	0.82	11812.71	7703.78	3.20E-03	0.65	0.49
N40-0.75	0.75	90.70	0.78	2930.86	3151.18	8.17E-03	1.08	0.77
H60-0.75		89.00	0.78	3146.42	3569.10	5.17E-03	1.13	0.82
S80-0.75		67.60	0.82	11471.78	13634.90	5.76E-03	1.19	0.86
N60-1.0	1.00	90.70	0.78	2930.86	3168.30	2.91E-03	1.08	0.67
H90-1.0		87.80	0.78	3117.68	3283.25	2.61E-03	1.05	0.65
S120-1.0		75.00	0.81	12250.50	12969.30	4.95E-03	1.06	0.86
N00-1.5	1.50	92.00	0.78	3217.27	2334.00	1.56E-03	0.73	0.38
N90-1.5		92.00	0.78	2961.38	3111.40	5.62E-03	1.05	0.71
H120-1.5		89.45	0.78	3157.14	3512.20	2.44E-03	1.11	0.63
S170-1.5		65.40	0.83	11233.68	13529.50	3.84E-03	1.20	0.79
S340-1.5		65.10	0.83	11200.98	11914.70	4.91E-03	1.06	0.73

**Table 4:** Design parameters and the comparison of TRs obtained from the experimental program and Equation (11).

Authors	*b* = *h* (mm)	*f*’_c_ (MPa)	Transverse Reinforcement Parameters					Steel Fiber Parameters			TR_hybrid_		
			*f_yt_* (MPa)	*ρ_h_*(%)	*k_e_*	*k_n_*	*T_It_* [Equation (3)]	*V_f_.l_f_/d_f_*	*τ_eq_* (MPa)	*T_If_* [Equation (5)]	pre ^(1)^	Exp ^(2)^	e (%) ^(3)^
Lima and Giongo (2004) [16]	150	68.00	656.00	0.61	0.11	2.00	0.33	40.00	5.80	3.41	0.56	0.57	−1.91
	150	68.00	656.00	1.82	0.44	2.00	3.87	80.00	5.80	6.82	0.70	0.78	−10.40
	150	91.00	656.00	1.82	0.44	2.00	2.89	80.00	6.67	5.87	0.67	0.71	−5.70
	150	91.00	656.00	1.82	0.44	2.00	2.89	80.00	6.67	5.87	0.67	0.70	−4.40
Paultre *et al.* (2010) [2]	235	91.30	428.00	3.29	0.78	1.33	9.01	50.00	9.80	5.37	0.75	0.66	13.50
	235	101.40	745.00	3.29	0.78	1.33	14.12	12.50	9.80	1.21	0.78	0.74	5.50
	235	99.50	410.00	3.15	0.67	1.33	6.51	50.00	9.80	4.92	0.72	0.70	3.47
Authors	200	90.70	478.00	7.92	0.53	2.00	11.15	59.25	9.05	5.91	0.78	0.77	1.33
	200	90.70	478.00	5.28	0.46	2.00	6.36	79.00	9.05	7.88	0.74	0.67	10.67
	200	92.00	478.00	3.52	0.35	2.00	3.23	118.50	9.05	11.66	0.73	0.71	2.70
	200	89.00	774.00	5.28	0.46	2.00	10.50	59.25	9.05	6.02	0.77	0.82	−5.71
	200	87.80	774.00	3.52	0.35	2.00	5.48	79.00	9.05	8.14	0.73	0.65	12.49
	200	89.45	774.00	2.64	0.26	2.00	3.00	118.50	9.05	11.99	0.73	0.63	15.71
	400	67.60	886.00	2.97	0.76	1.33	22.15	59.25	8.30	7.27	0.87	0.86	1.17
	400	75.00	886.00	1.98	0.65	1.33	11.43	79.00	8.30	8.74	0.80	0.86	−6.93
	400	65.40	886.00	1.40	0.53	1.33	7.52	118.50	8.30	15.04	0.80	0.79	0.94
	400	65.10	886.00	0.70	0.21	1.33	1.49	118.50	8.30	15.11	0.73	0.73	0.32

^(1)^ prediction. ^(2)^ experiment. ^(3)^ error (%)= (prediction/experiment−1) × 100%.

**Table 5:** Comparison study of the numerical model and experimental results.

ID	Curve	P_c,max_/P_o_	ε_max_	TR	TR Error (%)
S120-1.0	Experimental	1.06	4.95E-03	0.86	6.17
	Analytical	1.14	4.60E-03	0.81	
S170-1.5	Experimental	1.20	3.84E-03	0.79	5.95
	Analytical	1.05	5.44E-03	0.84	

## Appendix A: Example calculation

Consider reinforced concrete column S120-0.0 and S120-1.0, in which the detail section properties of these columns are summarized in Table 2, and the concrete compressive strength is based on a strength targeted of 70 MPa and the bar yield strengths are 685 MPa and 785 MPa for longitudinal and transverse bars, respectively. Determine the equivalent confinement from the column confined by transverse reinforcement and steel fibers with a fiber volume fraction of 1%.

## Solution:

**Equivalent confinement**:

Calculate k_n_ using Equation (4) and T_It_ using Equation (3):

(14) $$k_{n}=\frac{n_{l}}{n_{l}-2}=\frac{8}{8-2}=1.33$$

and

(15) $$T_{It}=\frac{k_{e}\cdot \rho_{h}\cdot f_{yh}}{f\prime_{c}k_{n}}=\frac{0.652\times 2\times 785}{70\times 1.33}=10.99$$

The equivalent bond strength $\tau_{eq}$ is calculated using micromechanical model proposed by Xu *et al.* [17]. The confinement index from steel fibers is:

(16) $$T_{If}=V_{f}\frac{L_{f}}{D_{f}}\times \frac{\tau_{eq}}{f\prime_{cf}}=1\times 79\times \frac{10.69}{70}=11.91$$

Next, calculate the toughness ratio TR column hybrid of transverse reinforcement and steel fibers using Equation (11):

(17) $$TR_{hybrid,pre}=0.48{\left(T_{It}+0.6T_{If}\right)}^{0.18}=0.48\times {\left(10.99+0.6\times 11.91\right)}^{0.18}=0.81$$

TR of 0.81 is substituted into Equation (3). Since the test results are provided, the *TR* of 0.86 (*TR* for S120-1.0 obtained from test result) is used in the next calculation.

Next, the *TR* of 0.86 is substituted into Equation (2) to compute the equivalent confinement index:

(18) $$0.86=0.48{\left(T_{It}\right)}^{0.18}$$

(19) TIt,equivalent=(0.860.48)(10.18)=25.53

Then, the equivalent volumetric ratio of transverse reinforcement in concrete core $\rho_{h}$ is obtained using Equation (3):

(20) $$25.53=\frac{0.652\times \rho_{h\_\text{eq}}\times 886}{75\times 1.33}$$

(21) $$\rho_{h\_eq}=4.41\%$$

The equivalent transverse reinforcement spacing is 120 mm×2%4.41%≈60 mm.
